# Efficacy of digital technologies aimed at enhancing emotion regulation skills: Literature review

**DOI:** 10.3389/fpsyt.2022.809332

**Published:** 2022-09-07

**Authors:** Ferozkhan Jadhakhan, Holly Blake, Danielle Hett, Steven Marwaha

**Affiliations:** ^1^Institute for Mental Health, School of Psychology, College of Life and Environmental Sciences, University of Birmingham, Birmingham, United Kingdom; ^2^School of Health Sciences, University of Nottingham, Nottingham, United Kingdom; ^3^NIHR Nottingham Biomedical Research Centre, Nottingham, United Kingdom; ^4^Birmingham and Solihull Mental Health NHS Foundation Trust, Birmingham, United Kingdom

**Keywords:** digital technologies, emotion regulation, mental disorders, wellbeing, intervention, adults, treatment

## Abstract

**Background:**

The impact of emotion regulation (ER) interventions on mental health and wellbeing has been extensively documented in the literature, although only recently have digital technologies been incorporated in intervention design. The aim of this review is to explore available published literature relating to the efficacy, barriers and facilitators of digital technologies in enhancing emotion/mood regulation skills.

**Methods:**

A review of the literature was performed to explore the effectiveness of digital technology in enhancing ER skills. MEDLINE, CINAHL, PsycINFO and Web of Science databases were searched from inception to 31st August 2020. In addition, the first 10 pages of Google Scholar were examined for relevant articles. The following MeSH term and key words were used to identify relevant articles: “emotion regulation OR mood regulation” AND “intervention OR treatment OR program$ OR therap$” AND “digital technologies OR web-based OR mobile application OR App.” Reference lists of retrieved papers were hand searched to identify additional publications. Findings were summarized narratively.

**Results:**

Titles and abstracts were reviewed by one reviewer in two phases, and confirmed by a second reviewer; discrepancies were resolved through discussion. First, the retrieved titles and abstracts were reviewed to identify relevant studies. Full texts of retrieved studies were then read to determine eligibility. The search resulted in 209 studies of which 191 citations were identified as potentially meeting the inclusion criteria. After reviewing the title and abstract of the 191 papers, 33 studies were identified as potentially meeting the inclusion criteria. Following full-text review, 10 studies met the inclusion criteria. Findings indicated the potential effectiveness of online, text-messaging, and smartphone interventions for enhancing ER skills.

**Conclusion:**

There is encouraging evidence that digital technologies may be beneficial for enhancing ER skills and providing personalized care remotely. Digital technologies, particularly the use of smartphones, were instrumental in facilitating assessments and delivering online self-help interventions such as cognitive behavioral therapy. Continued research is required to rigorously evaluate the effectiveness of digital technologies in ER skills and carefully consider risks/benefits while determining how emerging technologies might support the scale-up of ER skills and mental health treatment.

## Introduction

Digital technologies have become ubiquitous and integrated into everyday life, providing the potential to deliver mental health interventions more efficiently, while increasing accessibility (1). Since early 2020, to support the current public health response to the global coronavirus disease (COVID-19) pandemic, digital technologies are being harnessed at an unprecedented scale, for case identification, population surveillance and contact tracing (2).

Digital technologies include smartphones, virtual reality, wearable sensors, biofeedback techniques, web-based training programmes and mobile applications. Digital technologies generate, store and process numeric data using devices such as smartphones, computers, and multimedia (3). Interventions targeting ER using digital technologies have several advantages; namely, they provide researchers and clinicians with new platforms to offer support, monitor patient progress, as well as increasing patients' access to tailored treatment (4). Digital technologies are increasingly being used to manage and positively influence people's affective states including their emotions, mood, and stress levels. Several studies have found that digital technologies are a commonly used tool for ER, such as the use of videogames for relaxation, managing stress and controlling negative feelings (5). Social media and instant messaging services can provide a medium for ER, by regulating depressive emotions, reducing anxiety in stressful situations and/or reducing loneliness through social networking sites (6).

ER is a complex process which involves the ability to initiate, inhibit and regulate one's emotions in a given situation. ER is an integral part of how we feel, think about and experience our daily lives. ER is not aimed at eliminating emotions from our lives, but rather using them intelligently and flexibly to control their influence and produce desired responses to our mood (7). Several existing therapeutic approaches have been helpful with emotion regulation disorder. Such interventions are generally practical in nature and have a high success rate. One approach that is commonly used to help with ER is dialectical behavioral therapy (DBT). DBT is a type of cognitive behavioral therapy that focuses on enhancing ER and consists of individual therapies delivered by a trained therapist with occasional telephone follow-up consultations (8). Another approach involves acceptance-based behavioral therapy (ACT). This approach exposes individuals to experience problematic emotions in the context in which the function of language enhances the experience of unpleasant emotion in view to strip those emotions away. The focus of the treatment is facilitate individuals to adapt to those negative emotions and move toward a more fulfilling life (9). A further treatment approach is the mindfulness-based cognitive therapy (MBCT). This 8-week treatment programme targets emotion reactivity associated with stress by allowing individuals to break the dysfunctional cycle of rumination emotion regulation and self-criticism (10).

Digital technology can be used as a means to enhance positive emotion or help people to regulate their emotion at virtually any time and place, and this presents a unique opportunity to implement novel approaches or treatment mechanisms. Digital technologies allow for real-time measurement of cognitive, emotional, physiological, and behavioral responses in a variety of ‘real-world' situations while allowing for full experimental control (11). They can improve an individuals' ability to positively enhance their emotion skills and better manage mental health issues by training them to adopt contextually adaptive ER strategies. ER plays a key role in the development, maintenance, and treatment of various mental health problems (e.g., depression, borderline personality disorder, substance-use disorders, eating disorders, somatoform disorders) (12). Thus, the delivery of ER using digital technologies may be an important therapeutic target.

However, the efficacy and benefit of ER intervention using digital technologies to address mild to moderate and sub-threshold mental health conditions in adults is not yet established. The area of digital ER intervention has not been comprehensively reviewed to date. We therefore aimed to explore the published literature to better understand the efficacy of digital technologies in enhancing ER skills.

## Objectives

The objective of this review is threefold:

1. To summarize and map out the different types of ER interventions that are delivered using digital technologies.
2. To ascertain how diverse digital technologies can be applied to the delivery of ER interventions.
3. To explore barriers, facilitators, benefits, and caveats of using and implementing digital technologies in ER interventions (e.g., personalized care, blended treatment), and any impacts on clinical practice/services.

## Methods

### Search strategy

A literature search of the following databases MEDLINE, CINAHL, PsycINFO, and Web of Science was conducted from inception to 31st August 2020, and updated on 14th of October 2021. Additionally, the first 10 pages of Google Scholar were examined for relevant articles. The search included peer reviewed journals in the English language. The following MeSH terms and key words were used to identify relevant articles: (emotion regulation OR mood regulation) AND (intervention OR treatment OR program OR therapy) AND (digital technology OR web-based OR mobile application). A detailed Medline search strategy is provided (Supplementary Appendix 1). Reference lists of retrieved papers were manually searched to identify additional publications. The gray literature (opengrey.eu) was also searched to identify further relevant publications.

### Study selection

This review considered randomized controlled trials, cohort studies (retrospective and prospective), case-control studies (including nested case control), cross-sectional studies, feasibility studies and pre-post within-study designs. Included studies focused on ER for wellbeing utilizing digital technologies in adults with a diagnosed mental health disorder. Since we are interested in the potential value of ER interventions using digital technologies, papers considered eligible for inclusion in this review included:

1. Interventions using digital technology (e.g., including, but not limited to, smartphones, virtual reality, wearable sensors, biofeedback techniques, web-based training programmes and mobile Apps).
2. Data sources from personal devices which are for non-medical purposes (e.g., smartphones, digital watches).
3. Web services and passive modes of data collection which do not require any specific action or intervention from the user associated with enhancing ER.

English abstracts of non-English articles were reviewed where available. One reviewer (FJ) conducted screening and article identification and a second reviewer (SM) was consulted to resolve any uncertainties by discussion. Papers were excluded if they: (1) lacked the necessary information for review in the full-text or the abstract; (2) reported outcomes of studies with health conditions unrelated to mental health; (3) reported outcome data not related to digital technology data capture; (4) were studies including non-human participants. The titles and abstracts of reviews were identified, screened and classified for extraction of full review for further analysis. Results of the search are presented in Table 1 and the findings described narratively.

**Table 1:** Characteristics of included studies.

**Author**	**Year**	**Topic**	**Technology**	**ER module**	**Population**	**Sample size**	**Country**	**Study type**	**Intervention**	**Results**
**Mobile application**										
Leonard et al. (13)	2018	This study examined a mobile application that specifically aims at enhancing ER through the integration of data from self-reports and electrodermal activity	Mobile App and wearable sensor band	Extended Process Model of Emotion Regulation (EPMER)	Homeless adolescent mothers aged (13–21 years)	40	USA	Feasibility study	Mindfulness App *(Calm Mom)* combined with a sensor band	Overall acceptability rating = median 3.5 (range 3.3–3.8). Qualitative: Participants were the App benefitted them
Hides et al. (14)	2019	This study aimed to evaluate a new App called *Music eScape*, developed to assist young people with identifying, expressing, and managing emotions using music from their own music library	Mobile App	Difficulties in Emotion Regulation Scale (DERS)	Participants aged 16–25 years, who reported at least mild distress in the past month	169	Australia	RCT	Immediate vs. 1-month delayed access to *Music eScape*	No significant differences between immediate and delayed groups on DERS: baseline: mean = 7.66 (SD ±2.45); 6 months = 7.62 (SD ±2.72)
Morris et al. (15)	2010	This study examined the potential of mobile phone technologies to broaden access to cognitive behavioral therapy technique and to provide in the moment support	Mobile App	Mood map scale	Employees aged 30–48 years rated as having stress on the Mayo Health Risk Assessment	10	USA	Feasibility study	Mood map (via mobile App) prompting participants to report their moods several times a day	Participants reported improved anger, anxiety and sadness ratings (*p* <0.01)
Anand et al. (16)	2019	This study, used a smartphone application to monitor the effect of antidepressant on mood instability in terms of fluctuations between mania and depression, in young Major Depression Disorder (MDD) subjects who are at a high risk of developing Bipolar Disorder	Mobile App	Visual analog scale survey	Participants aged 15–30 years with bipolar disorder (BD) and unipolar major depression disorder (MDD)	40	USA	Case control study	Daily rating visual analog scale surveys capturing mood fluctuations	Daily ratings showed differences between groups (BD: 13%, HRMDD, 5%), (*p* = 0.02)
Porat et al. (17)	2020	This study aimed to test the feasibility of a mobile App ‘ReApp-, a mobile game designed to teach and train its users to regulate their emotions using cognitive reappraisal in participants exposed to the Israeli-Palestinian conflict	Mobile App	Emotion Regulation Questionnaire (ERQ)	Participants aged 29.8 years (SD ±9.6), exposed to conflict	70	Israel	Feasibility randomized controlled trial.	ReApp, mobile game	Results indicate that people who played ReApp experienced lower levels of anger (β = – 0.48, *p* <0.01) and disgust (β = −0.43, *p* <0.05)
Cikajlo et al. (18)	2016	This study evaluated a novel system for remote acquisition of a meditation-based stress and anxiety reduction therapy, mindfulness	Mobile App	(1) Mindfulness Awareness Scale (MAAS) (2) Mindfulness based stress reduction course (MBSR)	Participants aged 27–40 years with stress and anxiety	8	Slovenia	Cohort study	Mindfulness-based stress reduction VR Immersive head-mounted display Samsung Gear + Samsung Smartphone S6 and Note 4	MAAS mean scores reduced from 4.3 (±0.7) to 3.8 (±1.0). No significant difference in MBSR
**Web-based**										
Fonseca et al. (19)	2019	This study aims to evaluate a self-guided web-based intervention (Be a Mom) to prevent persistent post-partum depression in adult aged (≥18 years)	Web-based	Difficulties in Emotion Regulation Scale (DERS)	Participants aged 18–50 years in the early postpartum period	98	Portugal	A pilot randomized; two-arm controlled trial	Web-based self-guided cognitive-behavioral therapy intervention	Participants in the intervention group showed a significantly greater decrease in the levels of ER difficulties (*p* <0.001) and a significant greater increase in the levels of self-compassion (*p* <0.001) compared to the control group
Tsaousides et al. (20)	2017	This study sought to evaluate the efficacy of a Web-based group intervention (Online *EmReg)* to improve emotion regulation (ER) in individuals with traumatic brain injury (TBI)	Web-based	Difficulties in Emotion Regulation Scale (DERS)	Participants aged ≥18 years with Traumatic Brain Injury (TBI)	91	USA	Pre-/post-within-subject design with baseline, end-of-treatment, and 12-week follow-up assessments	Behavioral: Online Emotion Regulation Skills-training. 24 online emotional regulation skills-training sessions twice weekly and will complete online questionnaires sent every 4 weeks throughout baseline, the 12-week intervention, and 12-week follow-up	large effect size was found for the DERS at the 12-week follow-up (*d* = 0.65)
**Online**										
Addington et al. (21)	2019	The *MARIGOLD* (Mobile Affect Regulation Intervention with the Goal of Lowering Depression) study sought to test an online self-guided emotion regulation intervention for adults with elevated depressive symptoms	Online and mobile phone	Difficulties in Emotion Regulation Scale (DERS)	Participants ≥18 years with elevated depressive symptoms	137	USA	RCT	*MARIGOLD*, online self-guided positive emotion skills intervention	MARIGOLD participants showed significant increases in positive emotion over time, *b* = 0.12, *p* < .001, whereas positive emotion in the waitlist remained stable over time, *b* = 0.05, *p* = 0.36
**Virtual**										
Tong et al. (22)	2015	This study features an immersive virtual environment called “Virtual Meditative Walk.” The system Simulated walking meditation through a forest, applied as therapy for chronic pain management	Virtual reality (VR)	Numerical Rating Scale (NRS) for Self-Report Pain Levels (values 0–10)	Participants aged 35–55 years old with chronic pain	13	Canada	Case-control	The Virtual Meditative Walk *(VMW) +* biofeedback	These findings indicate that the VMW (VR paired with biofeedback for MBSR training) was significantly more effective than MBSR alone at reducing reported pain levels among participants

EPMR, extended process model of emotion regulation; VR, virtual reality; DER, Difficulties in Emotion Regulation Scale; NRS, Numeric Rating Scale; BD, bipolar disease; VMW, virtual meditative walk; MDD, major depressive disorder; MAAS, Mindfulness Awareness Scale; MBSR, mindfulness based stress reduction; MARIGOLD, mobile affect regulation intervention with the goal of lowering depression.

## Results

**Figure 1:**
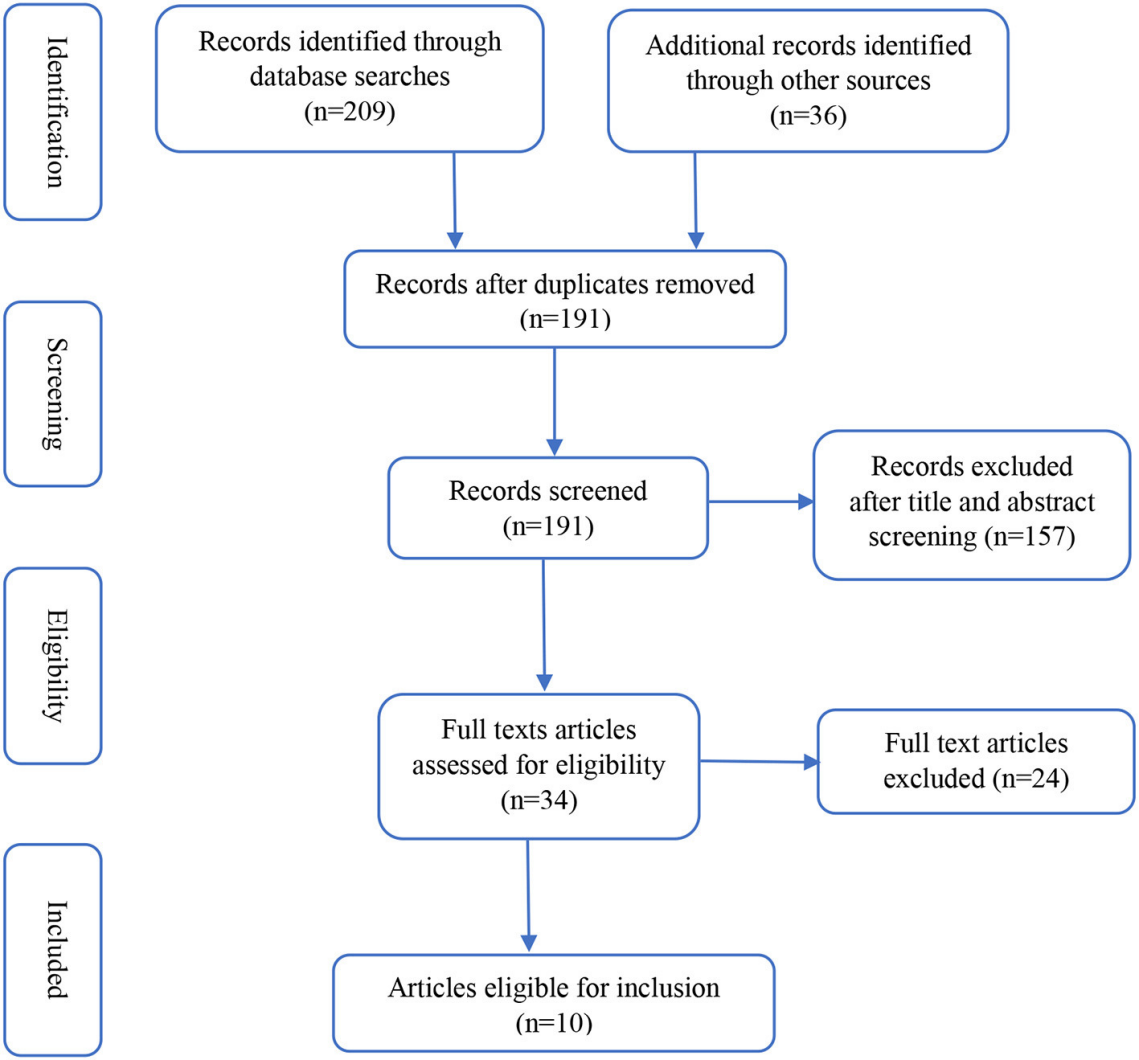
Results of literature review search strategy.

A record of the searches is provided in Figure 1. A total of 209 records were identified by database searches and a further 36 were sourced following the open gray database search. A further eight were retrieved by hand searching from references and one additional study was identified in the review update searches. Of the 209 studies, 18 were duplicates and the title and abstract of the remaining 191 studies were reviewed. Most exclusions were due to the article having no clear reference to ER intervention using digital technologies within the title or abstract. At this stage, 34 articles were further reviewed for inclusion, upon reviewing the full text of these studies, a further 23 were excluded specifically because they did not present ER interventions using digital technologies in an adult population. The remaining 10 studies were synthesized, a summary is provided in Table 1. Four randomized controlled trials (RCT) (14, 17, 19, 21), two feasibility studies (13, 15), two case-control studies (16, 22), 1 cohort study (18) and 1 pre-post within-study design (20) were identified. Five studies (13, 15, 16, 20, 21) were from the United States, one from Australia (14), one from Israel (17), and three from Slovenia (18), Portugal (19) and Canada (22) respectively.

A summary of the database searches that were performed during the process of conducting the review is set out below (Table 2). The search terms were reliable in identifying the most relevant articles related to digital technology relationship to ER.

**Table 2:** Medline database search strategy.

1	exercis*.mp
2	movement therapy.mp
3	exercise training.mp
4	physical activit*.mp
5	aerobic exercise.mp
6	physical exercise.mp
7	resistance training.mp
8	strength training.mp
9	endurance exercise.mp
10	cardiovascular exercise.mp
11	CV.mp
12	stretch*.mp
13	sport*.mp
14	PTSD.mp
15	exp. post traumatic stress
16	post traumatic stress disorder.mp
17	posttraumatic stress.mp
18	1–13 OR
19	14–17 OR
20	18–19 AND

### Mobile technologies

To date, only a few studies have examined the role of mobile applications in enhancing ER skills. These studies show that mobile phone technology usage varies according to types of intervention/approach and ER strategies. Six out of the 10 studies included in this review tested a psychological intervention module using different approaches such as social connection, text messages or a downloadable App. A mobile App is a type of application designed to run on mobile phones including smartphones or tablets/computer. Mobile health (mHealth) applications are contemporary approaches providing psychological support through mobile devices, without the involvement of a therapist or face-to-face intervention (10). mHealth allows the possibility of providing personalized interventions based on the current needs and affective state of the user. Many mHealth applications are mainly centered on cognitive behavior change (11), mindfulness (12) and cognitive behavioral therapy (CBT) (23). Morris et al. (15) examined the effect of self-reporting mood using the “Mobile Heart Health.” The programme involved using a *Mood Map* (charting mood for at least 1 week by identifying good, bad and indifferent mood) to self-report mood as well as physiological sensors to measure heart rate. Ten participants were recruited and indicated having significant stress but were otherwise mentally healthy. The aim of the study was to explore whether usage of this App would help reduce stress and related risk of cardiovascular disease. Participants' mood was assessed every morning, evening, and several times during the day over a month using the mood map App prompted via their mobile phones. Results showed a significant improvement in stress and a greater control over their emotions compared baseline measured by mood reporting scales (24).

A recent mHealth technology *(Calm Mom)* consisted of a mobile App and a wearable sensor band specifically aimed at enhancing ER through the integration of data from self-reports and electrodermal activity (variation of the electrical properties of the skin in response to sweat secretion) measured during adolescent mothers' daily lives. Both the quantitative and qualitative data showed that participants highly valued the accessibility of the *Calm Mom* App, both alone and in combination with the sensor band. For many adolescent mothers, the App became an integral part of the way in which they dealt with heightened emotions (e.g., fear, anger) in stress-inducing situations. The qualitative findings showed that the Calm Mom App helped to increase young mothers' understanding and ability to identify their emotions in a variety of stressful situations with their children, peers, and family, which in turn helped them engage in more adaptive ER and behavioral strategies (25). A recent study used a smartphone application to monitor daily and weekly mood ratings in young adults with bipolar disorder and healthy controls. Forty participants aged 18–30 years (*n* = 35 bipolar disorder; *n* = 5 control) were recruited and monitored for a total of 2401 daily and 744 weekly ratings. The researchers used The *Ginger IO* behavior platform to collect self-report data from participants (user input). The user receives notifications of available surveys and upon clicking they are transferred to the survey. The survey (visual analog scale) recorded time series scores collected from participants while under antidepressant treatment for mood instability. The primary outcome was to identify the number of survey ratings that can detect mood instability among Major Depression Disorder participants at high risk and those at low risk of developing bipolar disorder compared to healthy controls. A priory criterion to define mood instability included daily and weekly measurement of subthreshold hypomania scores in a clinical setting which consisted of a priori defined criteria for spikes in scores (>2 threshold and >25% increase from baseline) and fluctuations (change in two consecutive ratings of more than 25%).

Results showed that smartphone survey ratings can adequately capture mood instability in bipolar disorder and subjects at risk of major mood disorder compared to those at low risk of developing dipolar disorder and healthy controls (26). A study by Agyapong et al. (27) found that daily supportive text messages were a reliable and acceptable way of delivering adjunctive psychological interventions to people with mental health problems. *Text4Mood* is an innovative service designed to provide support to people on waiting lists for mental health services, or to individuals living in remote communities who have difficulty accessing care. Subscribers receive daily supportive text messages, for example:

1. Today, I will focus on what I have, instead of what I have not.
2. Within me lies the power to succeed.
3. I will work with what I have to reach my goals.

Upon receiving the text messages, 81.7% of users reported feeling hopeful in managing issues in their lives, 76.7% felt in charge of managing their depression and anxiety and 75.2% felt more connected to a support system. Text messaging could potentially be a useful service for delivery of remote care (e.g., in the context of a pandemic) to improve access to psychological care in a cost-effective and timely manner to individuals with a mental health issue who are unable to attend clinics (e.g., self-isolating). Short Messaging Service (SMS) mood monitoring has been validated against the Patient Health Questionnaire-9 (PHQ-9), a standardized measure of depression. Findings from a study by Aguilera et al. (28) demonstrated significant relationships between depression and daily mood scores, measured via SMS 1-week average mood scores and PHQ-9 scores captured on a daily basis controlling for linear change in depression scores. This study shows that SMS mood ratings could serve as a reliable proxy for in-clinic mood assessment (28).

Furthermore, Bush et al. (29) found that a smartphone application “*The Virtual Hope Box*” was more effective than print materials at improving military service men's ability to cope with negative emotions and thoughts and acceptability of treatment. Veterans in the study were receiving mental health treatment and had recently reported suicidal ideation. The App was modeled around a cognitive behavioral approach that uses a physical box containing various items which remind participants of positive experiences, reasons for living, coping mechanisms and people who care about them. Participants were randomly allocated to either the control (standard treatment and supplemental print materials) or intervention group (treatment as usual plus the *Virtual Hope Box* App). Compared to the control group, those assigned to the intervention group reported the App as being “somewhat helpful?” or “very helpful” (84%) and that they would be “somewhat” or “very likely” to use it again (87%) or recommend it to others (90%). The most frequent reasons for using the App were to cope with distress, overwhelming emotions, and thoughts of hurting themselves as well as for relaxation, distraction, or inspiration (29).

The *Mood Mission* App (30), developed by a team of researchers at Monash University (Australia), was designed to provide standalone help to individuals in response to how they are feeling at that time. The App was designed as a supplement to conventional face-to-face CBT. It provides the user with self-help strategies aimed at reducing feelings of anxiety and depression (30). This mobile App serves the purpose of preventing and coping with low level anxiety and depression. Results showed that users of *Mood Mission* experienced decreases in their depression and anxiety compared to baseline (30).

Another study aimed to evaluate a new App named *Music eScape* (14). This App aimed to teach young adults who were experiencing depression, anxiety and stress symptoms ways to identify and manage their emotions using music from their own music library. Participants were randomly assigned to either the “Tuned In” intervention (*eScape* mood management music App) or a wait-list control receiving the intervention after a 1-month delay. Participants in the intervention group were emailed a link to the App. The study evaluated ER, distress and well-being at 1, 2, 3- and 6-months intervals. Potential moderators to App outcomes, including the amount of music use and healthy (“protective” music which gives participants the energy to get going) or unhealthy (reminding participants of bad memories) music use, were examined. No significant differences between groups on ER, distress, or well-being were found at 1 month between groups. Both groups achieved significant improvements in 5 of the 6 ER skills, mental distress, and well-being at 2, 3, and 6 months compared to controls (14).

A study by Porat et al. (17) examined the feasibility of a mobile App “*ReApp*,” a mobile, multiplayer game aimed at training people in emotional regulation, specifically in cognitive reappraisal. Seventy Jewish-Israeli participants were randomly assigned to either an experimental (ReApp) or a control condition. Participants in the experimental condition played ReApp, while participants in the control condition played ‘*Connect Four'*, a two-player game played on a mobile device but which did not teach any cognitive skill. Results indicate that people who played ReApp experienced lower levels of anger and disgust compared to the control group.

Over the years, mindfulness-based interventions delivered via phone application have been examined. Tong et al. (22) showed mindfulness stress reduction in combination with virtual reality (virtual meditative walk) was significantly more effective in reducing reported levels of chronic pain and post-traumatic stress disorder compared to Mindfulness-Based Stress Reduction (MBSR) Meditation standalone. Furthermore, Cikajlo e al. (18) evaluated a novel system of a meditation-based stress and anxiety reduction therapy mindfulness and found that scores on both the Mindfulness Attention Awareness Scale (MAAS) and the Satisfaction with Life Scale (SWLS) improved after 8-weeks.

Researchers have reported that mindfulness-based smartphone App designed to assist people stop smoking was effective at reducing people's daily cigarette consumptions. Minami et al. (31) conducted a randomized controlled trail to assess the effectiveness of a smartphone assisted mindfulness intervention for smoking cessation in smokers receiving outpatient psychiatric treatment. Results showed that participants practiced mindfulness on average 3.4 times per day, completed 72% of prompted reports and submitted 68% carbon monoxide videos as requested. Participants reported that daily mindfulness practice was helpful for both managing mood and quitting smoking (31).

Additionally, Ying et al. (32) set out to examine the effectiveness of online mindfulness-based interventions on psychological distress (depression and anxiety). Seventy-six participants were randomized to either a group mindfulness-based intervention (GMBI), self-direct mindfulness-based intervention (SDMBI), discussion group (DG) or a control group (Blank Control Group). Results showed that participants in GMBI and SDMBI had significant pre- and post-test scores on mindfulness, ER difficulties, and psychological distress, with medium to large effect sizes in both groups.

Several studies have demonstrated the efficacy of App-based interventions to enhance ER. Morris et al. (15) developed an App that alert users to record their mood several times during the day over a month *via* their mobile phones. Users reported increased emotional self-awareness, and some were able to identify patterns of dysfunction and modify these patterns by modifying their routine. Anand et al. (16) used a smartphone self-monitoring programme to prompt users to complete a survey and report their emotional state on a weekly basis. Results showed that the mood ratings captured mood instability and offer a prudent way to monitor development of serious manic disorder. Both monitoring systems were, however modeled around the study participants and offered little constructive feedback about participant's mood history. A recent attempt using a mobile App specifically aimed at enhancing ER through the integration of self-reported data and electrodermal activity (13). Limitations of these studies were the small number of trial participants reporting mood disorders. There is a need to further investigate the impact of smartphone-based programme on larger more representative samples reporting ER and associated mental health outcomes which is also relevant to non-clinical users. Mobile phone technologies, in particular text messaging has transformed the way mental health interventions are delivered. Recent studies (27, 28) find text messaging interventions associated with reducing the stigma associated with mental health treatment and increase positive patient attitude and satisfaction as well as improved treatment adherence, increase appointment attendance (33). Despite high accessibility described in these studies, their applications were limited to patients with anxiety and depression, services such as addictions service delivery is relatively limited.

### Web-based interventions

Several studies have addressed the effectiveness of web-based and other computerized interventions in treating depression and anxiety symptoms (15, 23–26) but very few studies explored these technologies in supporting interventions to enhance ER. Internet technologies play a significant role in people's everyday lives especially social media and messaging App, and the remarkable uptake in video conferencing Apps, given people's widespread reliance of these technologies during the COVID-19 pandemic. Of the 10 studies included in this review, three reported web-based self-guided ER intervention, two were randomized controlled trials (RCT) and 1 was a pre-post within subject study. The use of the Internet an adaptation of face-to-face protocol is gradually becoming a favored platform in which ER plays an important role (34). A two-phase pilot project evaluating the *MARIGOLD* intervention (Mobile Affect Regulation Intervention with the Goal of Lowering Depression), a web-based, self-guided intervention based on “Stress and Coping” and “Broaden and Build” theories of positive emotion (21). The intervention was designed to teach individuals positive emotion skills for handling elevated depressive symptoms. Skills include noticing and amplifying positive events, gratitude, activation, mindfulness, positive reappraisal, strengths, and acts of kindness. Additionally, an internet-based ER training *(iERT)* aimed to enhance ER skills at reducing the risk of victimization in depressed patients is currently being investigated in a multicentre randomized controlled trial (35). Furthermore, researchers at the Traumatic Brain Injury Model System Center (New York, USA) enrolled 91 participants with traumatic brain injury in a study. Participants were enrolled in an experimental program, called Online *EmReg*, which was adapted from a face-to-face group therapy program. Over 12-weeks, participants received 24 1-h ER skills training sessions via videoconferencing, held twice a week for 12-weeks. The group sessions were held by experienced facilitators (i.e., rehabilitation neuropsychologists). The programme was designed to provide a learning platform for participants to learn how traumatic brain injury affects emotional functioning and provide strategies for improving ER skills in everyday life. During the final 16 programme sessions, participants practice their skills in individual and group exercises. At the end of the 12-week programme, participants showed meaningful improvements in ER on all aspects measured by the Difficulties in Emotion Regulation Scale (DERS) (20).

Motter et al. (36), in a double blind randomized controlled trial tested whether the Wechsler Adult Intelligence Scale focused computerized cognitive training programme (CCT) could out performed verbal ability focused CCT. This study aimed to explore the impact the cognitive functioning and mood of young adults with depressive symptoms. To test this, 46 young adults aged 18–29 years, with mild to moderate depression, were randomized to either the computerized cognitive training group or a verbal ability group. Participants trained on their mobile device 5 days per week for 8-weeks. Depressive severity, everyday functioning, and cognition were evaluated pre- and post-training. Results indicate that the computerized cognitive group had greater gains in executive functioning and processing speed than the verbal group. There were no di?erences between groups in mood or everyday functioning improvement.

Ouweneel et al. (37) evaluated the effect of an online positive psychology intervention to promote positive emotions, self-efficacy, and engagement at work. The online modules consist of three types of online assignments: happiness, goal setting, and resource building (intervention group) compared to a self-monitoring control group. Results revealed that the self-enhancement group showed a stronger increase in positive emotions and self-efficacy compared to the control group, but not on engagement. Additional analyses showed that the positive effects of the self-enhancement intervention are present for employees who are initially low in engagement (diary completion, survey, and online modules), but not for those medium or high in engagement.

Stubbings et al. (38) conducted a randomized controlled trial with 26, primarily Caucasian, adults aged 18–65 years. Participants were randomized to receive 12 sessions (60 min) of weekly sessions of CBT via videoconference (*n* = 14) or CBT delivered in person (*n* = 12) for participants with mood and anxiety disorders. Diagnoses were determined using the Diagnostic and Statistical Manual of Mental Disorders, 4th edition text revision (DSM-IV-TR). The Depression Anxiety Stress Scale (39) was used as the primary outcome measure. Significant reductions were found across time for symptoms of depression. The study found no significant differences between treatment conditions for any of the outcome measures.

Furthermore, Fonseca et al. (19) conducted a randomized controlled trial to test a self-guided, web-based CBT intervention to examine the feasibility, acceptability and efficacy of the “*Be a Mom*, web-based self-guided CBT intervention” an intervention aimed at preventing persistent post-partum depression in both at risk post-partum women and women presenting with early onset post-partum depressive symptoms. Eligible participants were either randomized to receive the intervention or randomized in a waitlist control group. Results showed a greater decrease in levels of ER difficulties in the intervention group and significant increased levels of self-compassion compared to the control group and significant decrease in depressive symptoms in the intervention group.

A wide range of interventions targeting ER has greatly benefited from incorporating online-based interventions. These recent developments include computerized (21) and (35) studies and videoconferencing technology (20). These technologies have a range of advantages, such as the possibility to reach larger audience in need of psychological treatment and hard to reach group, particularly young adults (40). In addition, online based interventions have increasingly been found to be cost effective in comparison to face-to-face active treatment (41), although some literature shows inconclusive results in this regard (42).

### Virtual environment

The rapid emergence of new technologies and the growing interest in applying them in the field of psychology have led to the development of novel virtual reality technology in the field of neuro-rehabilitation (43) and the treatment of various mental health disorders (44). Virtual reality uses computer technology to create a simulated environment in which a person can interact within an artificial three-dimensional environment using electronic devices such as head mounted display or biosensors to increase realism in the virtual world (13). To date, very few studies explored the potentiality of virtual reality impact on enhancing ER. Furthermore, the available studies are often small and conducted on specific populations. Evidence suggests that effective ER strategies lead to several important outcomes associated with mental health and psychological wellbeing (45). Therefore, beneficial changes in ER are crucial outcome in mental health interventions and the availability of new technology such as virtual reality technology can facilitate or even increase positive outcome of such strategies (46).

Tong et al. (22) showed that virtual meditation in combination with mindfulness-based stress reduction (MBSR) was significantly more effective in reducing reported levels of chronic pain (e.g., fibromyalgia) and post-traumatic stress disorder and a higher level of participants satisfaction and experience of higher level of satisfaction. This virtual reality system was designed for chronic pain patients to learn mindfulness based MBSR and incorporates biofeedback sensors, an immersive virtual environment and stereoscopic sound. Additionally, Cikajlo et al. (18) evaluated a novel system, the Realizing Collaborative Virtual Reality for Wellbeing and Self-Healing *(ReCoVR)* system for remote acquisition of a mediation based stress and anxiety reduction therapy and mindfulness course. The system is based on a cloud server web-interface and remote clients, using immersive head-mounted display Samsung Gear and Samsung Smartphone S6 and Note. The application enables group sessions in the virtual world, 3D videos and real-time interactions, as well as standalone meditation. An 8-week mindfulness-based stress reduction course was designed for this virtual reality application. The course was tested with 8 participants: 4 employees (age range 27–40 years) and four patients (age range 24–48 years) with traumatic brain injuries. Their outcomes were evaluated using the Mindful Attention Awareness Scale (MAAS), the Satisfaction with Life Scale (SWLS), and the Mini Mental State Examination (MMSE). The results were encouraging, patients achieved very high level of satisfaction (SWLS) at the end of the study. A slight increase in MASS score was also noticeable. Most patients had MMSE score of 30 suggesting normal cognitive function.

Although virtual reality (VR) is not new, the recent incorporation of such technology into ER treatment delivery is rather novel. In the studies presented, virtual reality has been utilized in various ways to deliver ER strategies to promote mental health and wellbeing. Some authors (18, 22) used mindfulness-based stress reduction intervention as a non-pharmacological approach to enhance participants ER and boost their wellbeing. Additionally, Tong et al. (22) showed that immersive virtual reality can be used to manage and control chronic pain in the long term.

## Discussion

This review aimed to investigate the available digital technologies that may be used to enhance ER skills and reduce the risk of mental health disorders or attenuate their effects. All the described digital technologies in this review were utilized to offer a different avenue of managing ER or enhance a current approach to improving knowledge about ER skills. Based on the reviewed studies, the current evidence suggests that most digital ER approaches lead to beneficial effects in reducing/enhancing ER by providing people with the ability/opportunity to regulate their emotions through enabling these practices at virtually any time and place. Only one study (14) did not report a significant difference between intervention and waitlist control on mean Difficulties in Emotion Regulation (DERS) scale at 1 month post intervention. Yet, both groups achieved significant improvements in 5 of the 6 ER skills, mental distress, and wellbeing at 2, 3, and 6 months compared to controls. However, other research suggests that some ER approaches can lead to poor outcomes based on how and when they are used (47), which highlights the need for future research to further explore issues of implementation and engagement. Our review included mostly feasibility studies and RCT's with small sample sizes and non-diverse populations, limiting the generalisability of the findings.

### Emotion regulation intervention delivered using digital technologies

In recent years, advances in information technology have facilitated the emergence of several digital mental health interventions, delivered via the internet, smart phones, virtual reality and videoconferencing. The wide use and availability of mobile technologies such as smartphones means that a wide range of people are able to access these technologies; this increases research opportunities in digital ER (34). Recent application of digital platforms to study ER has provided some fresh perspectives about this process (48) and a more complex representation and understanding of how people regulate their emotions (49). Considering the multi-faceted construct of health and well-being, this review provides an overview of the available literature and seeks to unravel the complex picture provided by the included studies. Overall, these studies show that digital technology is a promising method of delivering a group or individual intervention to improve ER following newly diagnosed or recurrent mental health disorders. Digital technologies can provide an effective alternative approach for diagnosis, assessment, and management of mental health disorders that appears to be comparable to in-person care. Furthermore, the connectivity, mobility and multi-functionality afforded by digital technologies provides greater access to a multitude of affordable technologies than ever before and the ease with which the user can navigate through apps allowing people to fine-tune their own strategies for particular situations and to engage in digital ER (50).

Five of the studies in this review applied mobile technologies in depressive disorder, traumatic brain injury ER and stress reduction (13–16, 21). Overall, most of the studies included in this review had design and/or methodological limitations (e.g., feasibility studies, no control group, small sample sizes). In addition, it is unclear which elements of the mobile application were the most beneficial, for example, the short therapies which were delivered in addition to the mobile application intervention, or the mobile application intervention itself as the mechanism for intervention delivery.

It will be of utmost importance to integrate dissemination and augmentation, particularly using mobile and ubiquitous technologies. For instance, there are some examples of integration, like VR through fully automated self-administered applications, which can be delivered remotely or digital augmentation through mobile technologies. This can represent an improvement for the clinical practice, either to increase the therapeutic content with the same amount of time with a therapist or decrease the time spent with the therapist but still having the same therapeutic content.

### Diverse digital technologies application in emotion regulation intervention

There is growing interest in the application of psychological interventions using digital technologies, likely because they are highly effective and scalable. This section describes some of these existing digital technologies used to enhance ER. Illustrative examples include web-based intervention such the Online *EmReg* (20) and *Be a Mom* mobile app (19) or the emerging field of virtual reality (18, 22). For instance, the Online *EmReg* has been shown to help people with traumatic brain injury improve their ER skills and reported high satisfaction with the intervention, as it provided treatment and support that these people would not normally have access to. Furthermore, virtual reality technique has been used to allow participants to learn mindfulness-based stress reduction technique and has been shown to significantly reduce pain in users (22). This goes to show that technological platforms such as web-based technologies and virtual reality can make evidence-based ER intervention that are currently conducted in therapy sessions more accessible and scalable with a far wider reach. Furthermore, advanced technological progress meant that seldom heard populations are now more accessible. For example, the “*Be a Mom”* intervention for postpartum women to prevent persistent postpartum depression symptoms (PPD) in at risk women presenting with early onset postpartum depressive symptoms. This self-guided web-based intervention grounded in cognitive behavioral therapy (CBT) has demonstrated a decrease in the levels of ER difficulties and a greater increase in in the levels of self-compassion in postpartum women receiving the “*Be a Mom*” intervention, compared to a control group (19).

### Barriers, facilitators, and potential benefit of digital technologies in emotion regulation intervention

This review identified some important facilitators and barriers to the use and implementation of digital emotion regulation. The main benefit of digital technology interventions targeting emotion regulation is greater dissemination of treatments (51), treatment customization and self-help treatments (52). Researchers have recently started to explore how individuals shape their affective states using digital technologies such as smartphones. The vast majority of digital emotion regulation approaches are in the form of cognitive behavior therapy. Most have been adapted from face-to-face treatment modalities; some are simplified versions of the original treatment whereas some combined both the treatment procedures and the strategies that govern their use. However, application of digital technologies has a number of benefits. Digital technologies make treatment more accessible, for example, CBT may be more feasible to deliver in remote and rural areas through widely used technology, such as smartphone. Furthermore, information gathered via digital technologies can be stored and accessed quickly and shared with patients and healthcare professionals. These data can be analyzed, and accurate reports can be derived making clinical decision-making easier. Additionally, digital technologies may lead to greater treatment adherence due to the accessibility of biofeedback participants receive in real time. “*Do-it-Yourself* ” (37) is an example of the positive effect of digital solution to promote positive emotion, self-efficacy and engagement at work that allow participants to complete online modules including happiness, goal setting and resource building while gathering biofeedback reports for healthcare professionals to always monitor progress. Although it is clear that digital technology has great potential within emotion regulation interventions, there are significant barriers preventing uptake of such mode of delivery. For example, digital literacy and accessibility barriers are often overlooked in the drive toward digital communication. Digital communication is effective only when it is inclusive in design - when recipients feel comfortable using technology with relation to their health, and when people of all abilities can access and understand the information being communicated. For example, some participants in the *MARIGOLD* study preferred to use pen and paper to record the study skill lessons instead of the *MARIGOLD* study website (21). There are technical considerations, for example, stable internet connectivity and in the case of mobile app, android with Bluetooth may be the only system supporting ER modules, which could be problematic for some participants to access. Most of the ER modules have only been designed and written in the English language limiting scalability across communities and geographical regions.

### Strength and limitations

This review has some limitations which should be acknowledged. The review primarily focuses on published articles, although gray literature and Google Scholar were searched, only articles published in the English language were reviewed, possibly missing peer reviewed papers published in other languages. Secondly, there is significant variation in the terminology of ER which may have led to omissions in our key words and MeSH terms. Thirdly, only 10 studies met our review criteria. The small number of available studies and the design and methodology limitations of included studies limits the potential for in-depth synthesis. The strength of this review includes searches across a broad range of medical databases, gray literature and Google Scholar and that study types were used to identify potential papers for inclusion. Our broad search strategy encompassed a wide spectrum of search terms, including emotion/mood regulation, digital technology, including mobile application, virtual reality, sensor bands and web-based psychological treatment modalities. The present review was broad in its approach because the main emphasis was on emotion regulation strategies using digital technologies, rather than the broader context of enhancing ER to better manage mental health disorders. The review considers individual differences in the digital approach used to enhance emotion regulation. For instance, if an emotion-regulation strategy is commonly used by certain individuals using a particular digital technology approach to target specific symptoms, highlighting this can bring into sharper focus the best treatment approach involved. Future studies may explore personalized treatment approaches targeting specific ER symptoms, as digital technology and assessments tools becomes more sophisticated so will our ability to provide better and more personalized treatment.

## Conclusion

Despite differing approaches that have been used to conceptualize ER and the use of various digital technologies to deliver complex ER strategies, ER using digital platforms has a broad and significant heuristic value in mental health research. The studies examined show that digital technology is likely to be an appropriate mechanisms for assisting individuals to enhance their ER skills and manage mental health issues better by accessing more adaptive ER strategies. The use of digital technology in this sense is promising because it allows the user to learn complex emotion regulation strategies in the context of life-like digital environments. For the aforementioned reasons, the pursuit of integrated prototypes of technologies could lead to a successful understanding, assessment, and training of emotion regulation and reduce the risk or better manage pre-existing mental health problems. The review highlights the importance of diverse digital technologies as platforms for enhancing emotion regulation and conveying individually tailored, emotional, physiological, and behavioral responses in a variety of ‘real-world' situations in ‘real-time'. There remains a need to explore which types of digital technologies provide better results, and why.

## Author contributions

FJ developed the search strategy, the review, and produced drafts of the manuscript. SM, HB, and DH provided input into the design and development of the manuscript. All authors contributed to and approved the final manuscript.

## Conflict of interest

The authors declare that the research was conducted in the absence of any commercial or financial relationships that could be construed as a potential conflict of interest.

## Publisher's note

All claims expressed in this article are solely those of the authors and do not necessarily represent those of their affiliated organizations, or those of the publisher, the editors and the reviewers. Any product that may be evaluated in this article, or claim that may be made by its manufacturer, is not guaranteed or endorsed by the publisher.

## Abbreviations

ER: emotion regulation
MARIGOLD: mobile affect regulation intervention with the goal of lowering depression
CBT: cognitive behavioral therapy
DSM: diagnostic and statistical manual of mental disorders
iERT: internet-based emotion regulation training
DERS: Difficulties in Emotion Regulation Scale
SWLS: Satisfaction With Life Scale
App: Application
MBSR: effects of mindfulness-based stress reduction
MAAS: The Mindful Attention and Awareness Scale
GMBI: group mindfulness-based intervention
SDMBI: self-direct mindfulness-based intervention
CCT: computerized cognitive training programme.
