# Supplementary material for: Efficacy of digital technologies aimed at enhancing emotion regulation skills: Literature review
Source: Front Psychiatry. 2022 Sep 7;13:809332. doi: 10.3389/fpsyt.2022.809332 (PMC9489858; doi:10.3389/fpsyt.2022.809332)
Supplement: Supplementary file 1 [file Data_Sheet_1.PDF]

Appendix 1: Medline search strategy

Part 1: Emotion regulation

1. Emotion regulation.mp
2. Emotion*.mp
3. Mood regulation.mp
4. Mood regulation*.mp
5. Affect.mp
6. Mental health wellbeing.mp

Part 2: Digital technology

1. Smartphone.mp
2. Smartphone*.mp
3. Wearable sonser*.mp
4. Biofeedback technique*.mp
5. Web based training*.mp
6. Social media.mp
7. Mobile application*.mp

Part 3: Intervention

1. Intervention*.mp
2. Treatment*.mp
3. Program$
4. Programme*.mp
5. Therap*.mp
